# Postoperative outcomes after receipt of ertapenem antimicrobial prophylaxis for colon surgery: a multicenter retrospective cohort study

**DOI:** 10.1017/ice.2024.99

**Published:** 2024-10

**Authors:** Christopher J. Hostler, Jay Krishnan, Alice Parish, Allison Baroco, Penny Cooper, Onofre Donceras, Ebbing Lautenbach, Pam Tolomeo, Tracy Sansossio, Carlos A.Q. Santos, David Schwartz, Helen Zhang, Sharon Welbel, Yuliya Lokhnygina, Deverick J. Anderson, for the CDC Prevention Epicenters Program

**Affiliations:** 1 Duke Center for Antimicrobial Stewardship and Infection Prevention, Duke University, Durham, NC, USA; 2 Durham VA Health Care System, Durham, NC, USA; 3 Duke Department of Bioinformatics and Biostatistics, Duke University, Durham, NC, USA; 4 Augusta Health, Fisherville, VA, USA; 5 John H. Stroger, Jr. Hospital of Cook County, Chicago, IL, USA; 6 Department of Medicine, Perelman School of Medicine, University of Pennsylvania, Philadelphia, PA, USA; 7 Division of Infectious Diseases, Department of Internal Medicine, Rush University Medical Center, Chicago, IL, USA

## Abstract

**Objective:**

To evaluate postoperative outcomes among patients undergoing colon surgery who receive perioperative prophylaxis with ertapenem compared to other antibiotic regimens.

**Design and setting:**

Multicenter retrospective cohort study among adults undergoing colon surgery in seven hospitals across three health systems from 1/1/2010 to 9/1/2015.

**Methods:**

Generalized linear mixed logistic regression models were applied to assess differential odds of select outcomes among patients who received perioperative prophylaxis with ertapenem compared to other regimens. Postoperative outcomes of interest included surgical site infection (SSI), *Clostridioides difficile* infection (CDI) and clinical culture positivity for carbapenem-resistant *Enterobacteraciae* (CRE). Inverse probability weights were applied to account for differing covariate distributions across ertapenem and non-ertapenem groups.

**Results:**

A total of 2,109 patients were included for analysis. The odds of postoperative SSI was 1.56 times higher among individuals who received ertapenem than among those receiving other perioperative antimicrobial prophylaxis regimens in our cohort (46 [3.5%] vs 20 [2.5%]; IPW-weighted OR 1.56, [95% CI, 1.08–2.26], *P* = .02). No statistically significant differences in odds of postoperative CDI (24 [1.8%] vs 16 [2.0%]; IPW-weighted OR 1.07 [95% CI, .68–1.68], *P* = .78) were observed between patients who received ertapenem prophylaxis compared to other regimens. Clinical CRE culture positivity was rare in both groups (.2%–.5%) and did not differ statistically.

**Conclusions:**

Ertapenem use for perioperative prophylaxis was associated with increased odds of SSI among patients undergoing colon surgery in our study population, though no differences in CDI or clinical CRE culture positivity were identified. Further study and replication of these findings are needed.

## Introduction

Over 300,000 colonic surgeries are performed each year in US acute care hospitals.^1^ Colon-related surgeries carry high morbidity, and some studies have cited up to 20% of patients develop surgical site infections (SSIs).^2,3^ These SSIs lead to significant adverse events; healthcare costs of SSIs range between $3.5 billion and $10 billion annually, and patients with SSI have 2–11 times increased risk of mortality compared to those without an SSI.^4^

Given the potential adverse events associated with SSI, hospitals use multiple strategies to reduce SSI risk. Guidelines from both national and international infection prevention organizations recommend the use of antimicrobial prophylaxis, which is the perioperative administration of an antibiotic with a spectrum of coverage effective against the most common pathogens encountered for a specific procedure.^4–6^ For surgical procedures involving the colon, prophylactic agents, such as cefazolin and metronidazole, cefoxitin, or ertapenem,^4^ are typically recommended along with oral antimicrobial prophylaxis.^5^

Antibiotic selection among recommended prophylaxis agents can be difficult and often involves discussions regarding differences in efficacy, logistics of administration, and potential side effects. Some published data have suggested ertapenem prophylaxis is more effective in the prevention of SSIs after abdominal surgeries compared to other regimens.^7,8^ In contrast, antimicrobial stewards have long worried about ertapenem’s broad spectrum of activity and potential for adverse effects. For example, some studies have noted an increased incidence of *C. difficile* infection (CDI) among those who receive ertapenem,^9^ while other studies have counterintuitively found reductions in carbapenem-resistant *Enterobacteriaceae* (CRE) rates with ertapenem prophylaxis.^10^

We performed this multicenter retrospective study of patients to evaluate postoperative outcomes among patients undergoing colon procedures who received perioperative prophylaxis with ertapenem in comparison with other antibiotic regimens.

## Methods

### Study design and ethics statement

We performed a multicenter, retrospective cohort study across seven hospitals in three health systems in both academic and community settings. We sought to test the *a priori* hypotheses that perioperative prophylaxis with ertapenem among patients undergoing colon surgery is associated with increased incidence of postoperative CDI, increased incidence of clinical CRE infection, and decreased SSI incidence in comparison with perioperative prophylaxis with other antimicrobial agents. Comparator prophylaxis groups included ampicillin-sulbactam, first- or second-generation cephalosporins, fluoroquinolones and clindamycin, third- or fourth-generation cephalosporins, or anti-pseudomonal penicillins. Other carbapenems were not included due to relative rarity of usage in perioperative prophylaxis settings compared to ertapenem among our study population.

This study was reviewed and approved by the Institutional Review Boards (IRBs) of all participating hospitals.

### Inclusion and exclusion criteria

All adult patients aged ≥18 years who underwent colon surgery at the study hospitals during the study period of 1/1/2010 through 9/1/2015 were included. *International Classification of Disease, Ninth Revision, Clinical Modification* (ICD-9-CM) codes were used to identify patients who underwent colon surgery. Only the first surgery during the study period for each patient who met criteria were included for analysis. Exclusion criteria included positive *C. difficile* test within 8 weeks prior to surgery date, positive CRE identification any time prior to surgery, lack of prescription of perioperative antibiotic prophylaxis, or infected wounds at time of procedure (as identified by either dirty wound class, infected wound class, or wound due to penetrating trauma as identified by ICD-9-CM codes [Supplemental Table 1]).

After initial data abstraction, we excluded two study hospitals because of incomplete data.

### Data collection

Data were abstracted from electronic medical records for all eligible patients among the study hospitals. Patient-specific data were obtained related to demographics, procedures, antimicrobial prophylaxis, and postsurgical outcomes, including postoperative CDI diagnoses, incidence of CRE positivity among clinical cultures (ie, excluding rectal screening cultures), SSI, and death. Participating hospitals also provided data related to baseline infection control data, such as hospital-wide and surgical-ward CDI and SSI rates as well as hand hygiene rates (Supplemental Table 2).

### Definitions

CDI was defined as a positive lab test for *C. difficile* toxin A and/or B tested on unformed stool specimen and/or a toxin-producing *C. difficile* organism as detected by PCR of an unformed stool specimen. A clinical CRE infection was defined as any positive clinical culture for *Enterobacteriaceae* organism resistant to imipenem, meropenem, doripenem, or ertapenem and/or documentation that the isolate possessed a carbapenemase gene by PCR; cultures obtained for screening purposes were excluded. SSI was defined according to 2020 Center for Disease Control NHSN surveillance criteria.^11^ Lastly, we define perioperative prophylaxis as use of antibiotics specifically for the prevention of SSI. Antibiotic treatment in the preoperative period was defined as receipt of any antibiotics prior to the surgical procedure, and antibiotic treatment in the postoperative period was defined as receipt of any antibiotics more than 24 hours after the surgical procedure, regardless of indication.

### Outcomes

The outcomes of interest were the cumulative incidence of CDI within 28 days of index colon surgery, the cumulative incidence of clinical CRE infection within 30 days of index surgical procedure, and the cumulative incidence of SSI within 30 days of index surgical procedure across eligible patients.

### Statistical analysis

Standard descriptive statistics were employed to describe hospital and patient demographics. To compare ertapenem and non-ertapenem groups, we fit generalized linear mixed models with a random intercept for hospital to estimate odds ratios for the incidence of postoperative CDI, clinical CRE infection, and SSI among those who received ertapenem versus other antimicrobial agents.

We calculated propensity scores and applied inverse probability weights (IPWs) to the models to control for observed differences between analysis groups for age, hospital, admission source (ie, nursing facility or not), preoperative length of stay, preoperative antibiotic treatment (ie, for indications other than prophylaxis), procedure duration, use of laparoscopy, procedure indication (ie, emergent or not), proton-pump inhibitor (PPI) use, and Charlson Comorbidity Index. Covariant balance was assessed to ensure appropriate balance was achieved after covariate weighting (Supplementary Table 3). Subgroup analyses were similarly performed to evaluate differences between the use of ertapenem and specific antibiotic agents or classes. Patients who received both ertapenem and one or more other antimicrobials in a comparator group were excluded from secondary analyses.

## Results

### Patient demographics and surgical characteristics

A total of 2,109 patients were included across five study hospitals, of which 1313 patients (62.3%) received ertapenem for perioperative prophylaxis (Table 1). Among 796 patients who received one or more non-ertapenem agents for antimicrobial prophylaxis, 376 patients (17.8%) received a first- or second-generation cephalosporin, 343 patients (16.3%) received a fluoroquinolone and clindamycin, 242 (11.5%) received an anti-pseudomonal penicillin, 16 (.8%) received a third- or fourth-generation cephalosporin, and 5 (.2%) received ampicillin-sulbactam. Note that some patients received multiple agents.

**Table 1.** Demographic and surgical characteristics of patients who received antimicrobial prophylaxis prior to colon surgery across five hospitals between 01/01/2010 and 09/01/2015 (n = 2109)

Metric	Ertapenem (n = 1313)	Non-Ertapenem (n = 796)
Age (median, IQR)	60 (49, 70)	62 (51, 72)
Sex (n, %)		
Female	727 (55)	453 (57)
Male	586 (45)	343 (43)
Race (n, %)*		
Asian/PI	10 (0.8)	5 (0.7)
Black	309 (24.6)	206 (27.0)
Native American	6 (0.5)	1 (0.1)
White	933 (74.2)	551 (72.2)
Ethnicity (n, %)*		
Hispanic/Latino	52 (4)	22 (3)
Not Hispanic/Latino	1189 (96)	722 (97)
BMI (median, IQR)	27 (23, 31)	27 (23, 31)
Charlson score (median, IQR)	2 (0, 6)	3 (1, 8)
Beta-lactam allergy (n, %)		
No	1177 (90)	576 (72)
Yes	136 (10)	220 (28)
PPI use (n, %)		
No	698 (53)	311 (39)
Yes	615 (47)	485 (61)
Antibiotic treatment in the preoperative period (non-prophylactic)*		
No	1098 (88)	489 (65)
Yes	152 (12)	68 (35)
Number of agents (median, IQR)	2.0 (1.0, 3.0)	2.5 (1.5, 4.5)
Antibiotic treatment in the postoperative period (non-prophylactic)		
No	714 (54)	142 (18)
Yes	599 (46)	654 (82)
Number of agents (median, IQR)	1.0 (1.0, 3.0)	3.0 (2.0, 4.0)
Admission source		
Acute care facility	48 (3.7)	82 (10.3)
Home/other	1260 (96.0)	705 (88.9%)
SNF/rehab	2 (0.2)	6 (0.8%)
Discharge destination**		
Acute care facility	20 (1.6)	30 (4.2)
Home/other	1075 (86.5)	501 (70.8)
SNF/rehab	138 (11.1)	114 (16.1)
Laparoscopic (n, %)		
No	1238 (94)	774 (97)
Yes	75 (6)	22 (3)
Emergent (n, %)		
No	810 (87)	330 (58)
Yes	125 (13)	242 (42)
Wound class (n, %)		
Clean	74 (7)	105 (16)
Clean contaminated	911 (83)	454 (70)
Contaminated	105 (10)	88 (14)
Unspecified	7 (0.6)	2 (0.3)
ASA score (n, %)		
1	8 (1)	5 (1)
2	225 (26)	97 (21)
3	557 (64)	259 (56)
4	78 (9)	100 (22)
5	1 (0.1)	5 (1)
Operation duration, minutes (median, IQR)	205 (137, 294)	170 (108, 256)

* Missing data included race (n = 88), ethnicity (n = 124), preoperative antibiotic administration (n = 102), admission source (n = 6) and discharge destination (n = 158).

** Excludes death (summarized in Table 2).

Several differences between analysis groups were observed. Patients who received ertapenem were more likely to have beta-lactam allergy but less likely to receive PPIs, non-prophylactic preoperative antibiotics, and non-prophylactic postoperative antibiotic treatment than patients in the non-ertapenem cohort (Table 1). Perioperative prophylaxis with ertapenem was noted more frequently among patients undergoing laparoscopy and non-emergent surgeries as well as among those with clean-contaminated wounds, generally lower ASA scores, and longer durations of surgeries (Table 1). Patients who received non-ertapenem antimicrobial prophylaxis were more likely to undergo emergency surgery and had higher mortality rates.

### Postoperative outcome assessment

Descriptive statistics regarding outcomes are provided in Table 2. A total of 66 patients were diagnosed as having SSI: 46 (3.5%) following receipt of ertapenem, and 20 (2.5%) following receipt of non-ertapenem antimicrobial prophylaxis. With unweighted analysis, no significant difference in the odds of postoperative SSI was observed among those who received ertapenem compared to other regimens in this cohort, though there did appear to be a trend toward worse outcomes with ertapenem (OR 1.74, [95% CI, 1.00–3.03], *P* = .05) (Table 3). Application of models with IPW demonstrated a statistically significant difference, however, where the odds of postoperative SSI was 1.56 times higher among individuals who received ertapenem than among those receiving other perioperative antimicrobial prophylaxis regimens (IPW-weighted OR 1.56, [95% CI, 1.08–2.26], *P* = .02) (Table 3).

**Table 2.** Descriptive statistics of postoperative outcomes among patients who received antimicrobial prophylaxis prior to colon surgery across five hospitals between 01/01/2010 and 09/01/2015 (n = 2109)

Outcomes by perioperative ertapenem use		
	Ertapenem n = 1313 (n, %)	Non-ertapenem n = 796 (n, %)
***C. difficile*** **infection (CDI)**		
No	1289 (98.2)	780 (98.0)
Yes	24 (1.8)	16 (2.0)
**Carbapenem-resistant** ***Enterobacterales*** **(CRE)**		
No	1311 (99.8)	792 (99.5)
Yes	2 (0.2)	4 (0.5)
**Surgical site infection**		
No	1267 (96.5)	776 (97.5)
Yes	46 (3.5)	20 (2.5)
**Multidrug-resistant organism**		
No	1305 (99.4)	779 (97.9)
Yes	8 (0.6)	17 (2.1)
**Readmission within 30 days**		
No	1238 (94.3)	757 (95.1)
Yes	75 (5.7)	39 (4.9)
**Death**		
No	1298 (98.9)	729 (91.6)
Yes	15 (1.1)	67 (8.4)

**Table 3.** Postoperative outcomes among patients who received antimicrobial prophylaxis with ertapenem compared to other antimicrobial regimens prior to colon surgery across five hospitals between 01/01/2010 and 09/01/2015 (n = 2109). Results from both unweighted and inverse propensity weighting (IPW) models are provided

Outcome (n)	Population size	Model	OR	95% CI		*P*-value
Surgical site infection						
Ertapenem (n = 46) versus All other perioperative prophylaxis (n = 20)	N = 2109	Unweighted	1.74	1.00	3.03	0.05
		Propensity IPW	1.56	1.08	2.26	0.02
Carbapenem-resistant *Enterobacterales* (CRE)*						
Ertapenem (n = 2) versus All other perioperative prophylaxis (n = 4)	N = 2109	Unweighted	0.35	0.06	1.96	0.23
*C. difficile* infection (CDI)*						
Ertapenem (n = 24) versus All other perioperative prophylaxis (n = 16)	N = 2109	Unweighted	1.00	0.52	1.92	0.99
		Propensity IPW	1.07	0.68	1.68	0.78
Ertapenem (n = 24) versus Ampicillin-sulbactam or first-/second-generation cephalosporin (n = 4)	N = 1503	Unweighted	1.47	0.50	4.36	0.48
		Propensity IPW	1.35	0.68	2.67	0.39
Ertapenem (n = 24) versus Fluoroquinolone and clindamycin (n = 4)	N = 1526	Unweighted	1.72	0.58	5.11	0.33
		Propensity IPW	2.31	1.05	5.10	0.04
Ertapenem (n = 24) versus Antipseudomonal Penicillin (n = 5)	N = 1487	Unweighted	0.97	0.36	2.66	0.96
		Propensity IPW	0.83	0.42	1.65	0.59

* Unweighted and propensity IPW Models were constructed to evaluate the impact of ertapenem versus third-/fourth-generation cephalosporins for C. difficile, but they did not converge as no cases occurred among third-/fourth-generation cephalosporins. Similarly, a propensity IPW model was constructed to evaluate the impact of ertapenem versus all other perioperative prophylaxis for CRE, but it did not converge.

Overall incidence of CRE positivity among clinical cultures (ie, those obtained for reasons other than screening) was low, with a total of two events occurring in the ertapenem group and four events in the non-ertapenem group (OR = .35 [95% CI, .06–1.96], *P* = .23; Table 3). Given the low frequency of the outcome, IPW models of postoperative CRE did not converge.

Forty patients had CDI: 24 (1.8%) cases of CDI occurred among patients who received ertapenem prophylaxis, while 16 cases (2.0%) occurred among patients who received other antimicrobial perioperative prophylaxis. No significant difference in postoperative CDI risk was observed between the ertapenem and non-ertapenem group with both unweighted (OR 1.00, CI, .52–1.92, *P* = .99) and IPW (OR 1.07, CI, .68–1.68, *P* = .78) analyses (Table 3). Although unweighted models did not demonstrate statistically significant differences between perioperative prophylaxis subgroups, application of IPW to our models found increased likelihood of CDI in the ertapenem group compared to those who received fluoroquinolones and clindamycin (OR = 2.31 [95% CI, 1.05–5.10], *P* = .04).

## Discussion

Clinical decisions regarding antimicrobial choices are generally made by weighing the expected benefit of the therapy against a variety of possible adverse outcomes. However, much of the existing literature on this topic is limited to examining the incidence of one to two adverse events after antimicrobial therapy, rather than examining multiple outcomes. To our knowledge, our study is among the few multicenter studies that evaluated CDI, CRE infection, and SSI by the type of antimicrobial prophylaxis. In our large cohort study of over 2,000 patients across multiple centers and practice settings, we observed an increased risk of postoperative SSI with the use of ertapenem compared to other regimens for antimicrobial prophylaxis prior to colon surgery; no major differences in the risk of postoperative CDI nor clinical CRE infection were observed. Taken together, these findings suggest that perioperative prophylaxis with ertapenem may be less preferable than other regimens based on its efficacy in the prevention of SSI in our cohort, though the incidence of the other studied postoperative outcomes of interest were similar.

Although unweighted analyses did not identify a significant difference in the odds of SSI among individuals who received ertapenem prophylaxis compared to other regimens, there was a notable trend toward increased SSI risk among ertapenem recipients. However, application of IPW models – weighted for variables including age, Charlson Comorbidity Index, and hospital for example – found that the odds of SSI with perioperative ertapenem was higher than that among individuals who received other regimens. These findings stand in opposition to prior data suggesting that ertapenem prophylaxis is more effective in the prevention of SSIs after abdominal surgeries compared to other perioperative prophylaxis regimens.^7,8^ This discrepancy will need further exploration in future studies, especially as unmeasured confounding (eg, specific indication type) could still confound the observed association between ertapenem and SSI in our cohort.

Incidence of postoperative CDI were generally similar among patients who received ertapenem compared to other antibiotics for perioperative prophylaxis with both unweighted and IPW analyses, with the exception of fluoroquinolone and clindamycin regimens in IPW models. We note that our overall findings conflict with other studies on the subject, which found more convincing relationships between ertapenem use and increased CDI incidence. For example, Lee et al^9^ found that the odds of CDI among patients receiving ertapenem prophylaxis was over three times the odds among those receiving other regimens in a case–-control study format.^9^ Our findings may be attributable to low overall CDI incidence among our cohort compounded with other factors unique to our study hospitals, which could include varying frequencies of surgical procedure type and baseline infection prevention/antimicrobial stewardship practices. Additionally, we excluded those with a positive *C. difficile* test within 8 weeks prior to surgery, which could have contributed to the low observed CDI incidence as well as potentially altered the association between ertapenem use and CDI incidence.

We did not observe a relationship between ertapenem use for perioperative prophylaxis and clinical CRE infection compared to other regimens. Low incidence of postoperative CRE clinical culture positivity were observed among our cohort, which limited our ability to apply IPW models and draw further inference. However, it should be noted that carbapenem exposure is not as strongly linked to CRE compared to CDI, as prior studies have even suggested an inverse relationship between ertapenem surgical prophylaxis and CRE.^10^

Our study has several limitations. First, the occurrences of CDI, CRE infection, and SSI were low despite a large cohort These low event rates significantly limited the power of our study. Statistical test results must be interpreted with caution because of decreased power and as we did not correct for multiple hypothesis testing. Second, data about duration of postoperative antibiotic therapy and/or concurrent oral antibiotic receipt for prophylaxis were not available for our study population. Given a substantial number of participants in the non-ertapenem group comparatively received postoperative antimicrobials, it is possible that duration of antimicrobial therapy drove the observed group differences. Similarly, choice of concurrent oral antimicrobial could impact results. Third, the indications for ertapenem use varied both across and within institutions. This potential selection bias is likely demonstrated in the demographic differences noted between ertapenem and non-ertapenem prophylaxis groups (eg, ertapenem was more commonly used in non-emergent procedures). In addition to our large study sample, we attempted to mitigate the impact of this limitation by controlling for potential differences across hospitals using propensity scores. Fourth, the analysis of CRE and CDI was also limited to identification within 30 days and 28 days postoperatively, whereas changes in gut microbiome have been noted to persist even 2 years after antibiotic exposure.^12^ More study of ertapenem prophylaxis should be pursued for longer study periods among institutions with high rates of CDI or CRE infection and among patients undergoing surgeries beyond colonic procedures. Finally, our study data were derived from 2010 to 2015, which may partly limit generalization of our findings to more recent settings where laparoscopic procedures are more prevalent, for example. Overall, in light of these limitations and the inherent limitations of our retrospective study design, our findings should primarily be used for hypothesis generation and guidance for future study.

In summary, in our large multicenter cohort study, ertapenem use for perioperative prophylaxis prior to colon surgery was associated with increased odds of SSI compared to other antimicrobial prophylaxis regimens, though no substantial differences were noted with CDI or clinical CRE infection. Prospective evaluation using randomization may be required to overcome potential issues related to selection bias to validate our findings. Altogether, these findings underscore the need for further study and compare specific agents for antimicrobial perioperative prophylaxis.

## Supporting information

Hostler et al. supplementary material Hostler et al. supplementary material

## Supplementary material

To view supplementary material for this article, please visit https://doi.org/10.1017/ice.2024.99
