# Supplementary material for: Postoperative outcomes after receipt of ertapenem antimicrobial prophylaxis for colon surgery: a multicenter retrospective cohort study
Source: Infect Control Hosp Epidemiol. 2024 Oct 4;45(10):1162–7. doi: 10.1017/ice.2024.99 (PMC11611502; doi:10.1017/ice.2024.99)
Supplement: Hostler et al. supplementary material [file S0899823X24000990sup001.docx]

**Supplemental Table 1:** ICD-9 codes and hospital surgery codes used to identify colon surgeries at participating hospitals.

| **ICD-9-CM Principal Procedure Codes for Study Inclusion** | |
| --- | --- |
| **ICD9 Code** | **ICD9 Code Description** |
| 45.03 | Incision of large intestine |
| 45.49 | Other destruction of lesion of large intestine |
| 45.71 | Multiple segmental resection of large intestine |
| 45.72 | Cecectomy |
| 45.73 | Right hemicolectomy |
| 45.74 | Resection of transverse colon |
| 45.75 | Left hemicolectomy |
| 45.76 | Sigmoidectomy |
| 45.79 | Other partial excision of large intestine |
| 45.8 | Total intra-abdominal colectomy |
| 45.92 | Anastomosis of small intestine to rectal stump |
| 45.93 | Other small-to-large intestinal anastomosis |
| 45.94 | Large-to-large intestinal anastomosis |
| 45.95 | Anastomosis to anus |
| 46.03 | Exteriorization of large intestine |
| 46.04 | Resection of exteriorized segment of colon |
| 46.10 | Colostomy, not otherwise specified |
| 46.13 | Permanent colostomy |
| 46.75 | Suture of laceration of large intestine |
| 46.76 | Closure of fistula of large intestine |
| 46.94 | Revision of anastomosis of large intestine |

One hospital (hospital 5) did not list procedures as ICD codes, but rather by a descriptive name. The following names were used in this study.

| Abdominal Bowel Resection |
| --- |
| Abdominal Colectomy |
| Abdominal Colectomy Laparoscopic |
| Abdominal Colectomy Sigmoid |
| Abdominal Colostomy Construction Laparoscopic |
| Abdominal Colostomy Revision |
| Abdominal Colostomy Transverse Loop |
| Abdominal Exploratory Laparotomy |
| Abdominal Hemi Colectomy |
| Abdominal Hemi Colectomy Laparoscopic |
| Abdominal Proctocolectomy |
| Abdominal Sigmoid Colon Resection |
| Abdominal Sigmoid Colon Resection Laparoscopic |
| Abdominal Sigmoidectomy |
| Anterior and Posterior Repair |
| Colon Resection |
| Colon Resection Laparoscopic |
| Colon Resection Low Anterior |
| Colostomy Construction |
| Excision Polyp Rectal |
| Excision Rectal Tumor Trans Anal |
| Hemicolectomy |
| Ostomy Construction Laparoscopic |
| Rectopexy |
| Resection Low Anterior Colon |
| Revision Colostomy Laparoscopic |
| Sigmoid Colon Resection |
| Takedown Ostomy |
| Takedown Ostomy Laparoscopic |

**Supplemental Table 2:** Infection control metrics of participating hospitals

| Hospital | Procedures  N (% of total cohort) | % received ertapenem | Average Number of Patient Days per Month | Hospital HO-CDI rate per 1000 | Hospital CA-CDI rate per 1000 | Hospital hand hygiene rate | Timeframe of data included |
| --- | --- | --- | --- | --- | --- | --- | --- |
| 1 | 102 (4.8) | 63 (61.8) | 3697 | 0.565 | 2.716 | 0.651 | 08/01/13 - 09/01/15 |
| 2 | 1264 (59.9) | 814 (64.4) | 22572 | 0.783 | 0.602 | 0.898 | 01/01/10 - 09/01/15 |
| 3 | 602 (28.5) | 378 (62.8) | 9094 | 1.567 | 1.825 | 0.825 | 01/01/10 - 09/01/15 |
| 4 | 65 (3.1) | 12 (18.5) | 5316 | 0.853 | 1.815 | 0.875 | 01/01/10 - 09/01/15 |
| 5 | 76 (3.6) | 46 (60.5) | 3188 | 0.730 | 1.153 | 0.951 | 01/01/10 - 09/01/15 |

**Supplemental Table 3:** Covariate balance table for propensity scores (PS)

| Covariate Name | Test Statistic Before PS Adjustment | P Value for Test Before PS Adjustment | Test Statistic After Inverse Weighting | P Value for Test After Inverse Weighting | DF for Test |
| --- | --- | --- | --- | --- | --- |
| Age | 6.704 | 0.010 | 0.023 | 0.879 | 1.000 |
| Charlson Score | 22.906 | <0.001 | 0.101 | 0.751 | 1.000 |
| Hospital ID | 55.705 | <0.001 | 3.557 | 0.469 | 4.000 |
| Laparoscopic procedure | 9.817 | 0.002 | 0.323 | 0.570 | 1.000 |
| Operation duration | 24.795 | <0.001 | 0.022 | 0.882 | 1.000 |
| Receipt of PPI | 39.429 | <0.001 | 0.279 | 0.597 | 1.000 |
| Antimicrobial prophylaxis agent | 151.648 | <0.001 | 0.073 | 0.787 | 1.000 |
| Pre-operative length of stay | 53.957 | <0.001 | 0.183 | 0.669 | 1.000 |
